# The short-term efficacy of low-frequency rTMS versus continuous theta burst stimulation as early augmentation, targeting right DLPFC in the management of obsessive-compulsive disorder: a randomized clinical study

**DOI:** 10.1017/S1092852925100527

**Published:** 2025-08-26

**Authors:** Vishal Kumar Gautam, Tapas Kumar Aich, Amil Hayat Khan, Sujita Kumar Kar, Ajeet Chaudhury, Umashankar Kushwaha

**Affiliations:** 1 Department of Psychiatry, BRD Medical College, Gorakhpur, UP, India; 2 Department of Psychiatry, King George’s Medical University, Lucknow, UP, India

**Keywords:** Obsessive-compulsive disorder, low-frequency rTMS, continuous theta burst stimulation, efficacy, early augmentation

## Abstract

**Background:**

Obsessive-compulsive disorder (OCD) is a significantly disabling and difficult-to-treat psychiatric disorder. Non-invasive neuromodulation techniques like repetitive transcranial magnetic stimulation (rTMS) have been increasingly used in the management of OCD. This study aimed to compare the efficacy of early augmentation with low-frequency rTMS (LF-rTMS) and continuous theta burst stimulation (cTBS) in improving psychopathology in OCD patients.

**Methods:**

The study design was a parallel-group, double-blind, randomized clinical trial. The study recruited 46 OCD patients who were randomly allocated to receive either LF-rTMS or cTBS (23 patients in each group) following the computer-generated random table method. All participants were rated on YBOCS, HAM-A, and HAM-D at baseline and third week and sixth weeks. These patients received a total of 15 sessions of LF-rTMS or cTBS stimulation once daily for 5 consecutive days in a week for 3 consecutive weeks over the right dorso-lateral pre-frontal cortex (DLPFC) area.

**Results:**

There was a statistically significant improvement in the total YBOCS score for both the LF-rTMS group and the cTBS group at the end of the third and sixth week when compared with their baseline scores. However, there was no statistically significant difference between the 2 groups in terms of the improvement in the total YBOCS score, as well as the total scores for the HAM-A and HAM-D during the follow-up periods.

**Conclusion:**

The study results suggest that both LF-rTMS and cTBS were equally effective in managing OCD patients as an early augmentation strategy.

## Introduction

Obsessive-compulsive disorder (OCD) is a chronic disabling neuropsychiatric disorder. Worldwide the lifetime prevalence of OCD ranges from 1% to 3% with a higher rate (2–3%) in developed countries.^1^ ^,^^2^ Since the introduction of selective serotonin reuptake inhibitors (SSRIs) in the 1980s, the treatment of OCD has dramatically improved. Evidence of combined pharmacological (SSRI) and cognitive behavior therapy (CBT) are better than either of the treatment alone.^3^ Unfortunately, a significant proportion of patients with OCD (ranging from 40% to 50%), fail to respond with the currently available first-line treatment strategies.^3^ ^,^^4^

Repetitive transcranial magnetic stimulation (rTMS) is a novel, safe, non-invasive brain stimulation technique that uses repetitive, brief, intense magnetic fields generated by a coil placed over the scalp, producing an electric field in the underlying brain region through electromagnetic induction.^5^ ^,^^6^

Neurobiological models of OCD describe alterations in cortico-striato-thalamo-cortical (CSTC) circuits responsible for affective, cognitive, and motor functions. Anterior cingulate cortex (ACC) and medial prefrontal cortex (mPFC) are key components of this loop, whose dysfunction has been consistently reported in neuroimaging studies.^7^ Neurophysiological studies reveal that the dorsolateral prefrontal cortex (DLPFC), supplementary motor area (SMA), and orbitofrontal cortex (OFC) are hyperactive in patients with OCD, and this hyperactivity has been associated with deficits in processing information and response control.^8^

Different neural structures implicated in the pathophysiology of OCD have been targeted using various brain stimulation methods. As rTMS can modulate cortical activity, it has been utilized in the treatment of OCD. The heterogeneity in protocols and conflicting results in the limited literature on rTMS in OCD have made it difficult to conclude whether rTMS is efficacious from individual studies, but meta-analyses have revealed that active rTMS is superior to sham rTMS in the treatment of OCD.^9^

Over the last decade, theta burst stimulation (TBS) has been increasingly used as an experimental and therapeutic tool. TBS is a form of repetitive transcranial magnetic stimulation (rTMS) involving the use of triple-pulse bursts at 50 Hz repeated 5 times per second (every 200 milliseconds, 5 Hz) in either a continuous (cTBS) or intermittent fashion (iTBS).^10^

The application of TBS to the treatment of psychiatric disorders is relatively new and mostly limited to major depression. To date, there is very few experimental data or double-blind randomized controlled trials on the effectiveness of TBS for reducing clinical symptoms in OCD patients. Again, in the management of OCD, the supplementary motor area, pre-supplementary area, left DLPFC, and orbitofrontal cortex are targeted in most of the research involving non-invasive neuromodulation techniques.^5^ ^,^^9^ ^,^^11^ ^–^^14^ In the recent years, the right DLPFC has been targeted using low-frequency rTMS (LF rTMS) and found to be effective in improving symptoms of OCD.^15^ ^–^^18^ Continuous theta burst stimulation (cTBS) of the right DLPFC was found to reduce the impulsive decision making.^19^ cTBS targeting the right DLPFC was also found to reduce the symptoms of OCD^20^ ^–^^22^; however, the findings are inconsistent, and the number of studies is few. After an extensive literature search, we did not find any study that compared the efficacy of conventional LF-rTMS with cTBS over the right DLPFC. Hence, the current study was planned to compare the efficacy of LF-rTMS and cTBS at the right DLPFC in reducing anxiety, depressive, and OC symptoms in OCD patients as an early add-on treatment.

## Materials and method

The study was conducted at the department of psychiatry in a tertiary care hospital in the eastern part of Uttar Pradesh, India. It was a parallel-group, double-blind, randomized clinical trial. The sampling frame was all OCD patients who attended our psychiatry outpatient department (OPD) during the period of January 2023 to December 2023. Randomization of groups was done using a computer-generated random number method. The sample size was estimated using G*Power software, taking the following variables into consideration:

**Table:** 

**F tests -** ANOVA: Repeated measures, within-between interaction			
**Analysis:** A priori: Compute required sample size			
**Input:**	Effect size f	=	0.25
	α err prob	=	0.05
	Power (1-β err prob)	=	0.90
	Number of groups	=	2
	Number of measurements	=	3
	Corr among rep measures	=	0.5
	Nonsphericity correction ε	=	1
**Output:**	Noncentrality parameter λ	=	13.5000000
	Critical F	=	3.1316720
	Numerator df	=	2.0000000
	Denominator df	=	68.0000000
	Total sample size	=	36
	Actual power	=	0.9060304

A dropout of 20% was considered, which made the target sample to 45, which was rounded off to 46. As a result, the study’s final sample consisted of 46 OCD cases, 23 each of which were in the groups for LF-rTMS and cTBS.

For enrolment to the study, the inclusion criteria were the diagnosis of obsessive-compulsive disorder using Diagnostic Criteria for Research (DCR) of ICD 10, a Y-BOCS score of ≥16, age between 18 and 60 years, either sex, right-handed, and all those patients who gave written informed consent to participate in the study.

Exclusion criteria were the patients with co-morbid neurological or any other psychiatric disorders (except mild-to-moderate depression, HAM-D score 18 or less), comorbid active substance use (apart from tobacco use disorder), patients undergoing concomitant cognitive behavior therapy, and those with unstable medical conditions, pregnancy or breastfeeding, having past history of epilepsy, significant head injury, any neurosurgical procedure, patients who cannot undergo TMS procedure due to presence of contraindications for it (having cardiac pacemaker or aneurysmal clip or cochlear implant or ventriculo-peritoneal shunting) and the patients who had received electroconvulsive therapy in past 6 months for any indication.

Study tools used for the study were informed consent and patient’s instruction form, a specially designed socio-demographic and clinical data sheet used for the present study, DCR of ICD-10,^23^ Handedness preference schedule,^24^ Hamilton Depression Rating Scale (HAM-D),^25^ Hamilton Anxiety Rating Scale (HAM-A),^26^ Yale-Brown Obsessive Compulsive Scale (Y-BOCS),^27^ and rTMS side-effects checklist.^6^ A brief description of some of these instruments is as follows:

Handedness preference: The Hindi version of this tool was used to assess the handedness among the participants. The questionnaire contains 15 activities, each rated by the participants based on their hand preference on a 5-point reporting scale for each activity.^24^

HAM-D: The 17-item version of the HAM-D has been used for the present study.^25^ Each of the 17 items are scored on a severity scale of 0 (not present) to 4 (severe). The total HAM-D score is obtained by adding the score of each item. Scores can range from 0 to 54 for the 17-item version of the scale. Total scores of 0–6 do not indicate the presence of depression, scores of 7–17 represent mild depression, scores of 18–24 represent moderate depression, and scores more than 24 indicates severe depression. A clinically significant change is usually defined as a decrease of 50% or more from baseline during the treatment.

HAM-A: The HAM-A is one of the most widely used rating scales to measure the severity of anxiety symptoms in clinical practice and research settings. The scale consists of 14 items, each defined by a series of symptoms. It measures both psychic anxiety (mental agitation and psychological distress) and somatic anxiety (physical complaints related to anxiety). Each of the 14 items are scored on a severity scale of 0 (not present) to 4(severe). The total score ranges from 0 to 56, where a score 30 and above indicates severe anxiety.^26^

Y-BOCS: OCD assessment begins by administering the YBOCS symptom checklist interview, which is a 64-item clinician administered checklist that examines present and past obsessions and compulsion. A clinician-administered semi structured scale is used to measure the severity of obsessions and compulsions. This is a 10-item scale with 5-point scale ranging from 0 (no symptoms) to 4 (extremely severe symptoms). There are 5 items each for obsessions and compulsions namely (viz.,) time spent, interference, distress, resistance, and control. However, there is no scoring for the checklist. The total score for this severity scale range between 0 and 40 which represent the total symptom severity. Additionally, scores for items 1–5 can be added to obtain a sub-score for severity of obsessions and scores for items 6–10 can be added to obtain a sub-score for severity compulsions.^27^ A total score ≥ 16 on the YBOCS is the cut-off usually used in therapeutic trials to identify clinically symptomatic levels of OCD ^27^. Total scores between 0 and 7 are considered to be indicative of subclinical OCD symptoms, 8–15 represents mild, 16–23 represents moderate, 24–31 represents severe, and 32–40 indicates extreme severity.^27^

rTMS Side Effects Checklist: This includes the most common side effects of rTMS. It was administered at the end of each session of rTMS to ascertain the side effects, if any, and their severity.^6^

## Procedure

Patients aged ≥18 years attending the Psychiatry OPD during the time period from January 2023 to December 2023, diagnosed with OCD by a psychiatrist as per ICD-10 criteria, were screened as per the inclusion and exclusion criteria. All the recruited OCD patients were without the characteristic of treatment resistance. A written informed consent was taken from all the participants. The socio-demographic and clinical details were recorded on the semi-structured proforma. All study participants were randomly assigned into Group A (LF-rTMS) and Group B (cTBS) by using computer-generated random number methods, and the primary investigator, outcome assessors and the patients were kept blinded to treatment arms.

All the patients included in the study were drug-naïve at the time of initial assessment and were started on 40 mg/day fluoxetine as part of a standardized treatment protocol designed for this trial for at least 2 weeks prior to the first session of rTMS. Using fluoxetine as a consistent SSRI across all participants allowed us to ensure homogeneity in baseline pharmacological exposure. Both groups received the ongoing treatment at the same dose during the study period also. Each study subjects were assessed by applying “Handedness Preference Schedule,” “Yale Brown Obsessive Compulsive Scale,” “Hamilton Rating Scale for Depression (HAM-D),” and “Hamilton Rating Scale for Anxiety (HAM-A)” at the time of first assessment. All psychometric assessment performed by same psychiatrist at different timepoints in each patient during the study period.

Resting motor threshold (RMT) measurement was done by thumb movement visualization method by stimulating the non-dominant primary motor cortex. Similar coils were used for both the groups for identification of RMT. Study participants of both groups were given either LF-rTMS or cTBS by double blind method with the use of morphologically similar coils with following the standard protocol of TMS in OCD. The right dorsolateral prefrontal cortex (DLPFC) area was chosen as the site of stimulation. The 5 centime rule was used for localization of the DLPFC. In both groups, total 15 stimulation sessions were given once daily for 5 consecutive days in a week for 3 consecutive weeks using Magstim Rapid device (The Magstim Company Limited, Whitland, UK) with a focal 70-mm figure 8-shaped coil.

In the LF-rTMS group, in each session, participants were delivered 20 trains of 80-second train width, each separated by 5-second intervals, using 1 Hz frequency at 100% of RMT, which accounted for a total of 1600 pulses per session and required 28 minutes for each session. In the cTBS group, study subjects were administered cTBS stimulation at 100% of RMT, during which 3 single biphasic pulses separated by 0.02 s (50 Hz) repeated every 0.2 s (5 Hz) for a total of 600 pulses were delivered in a 40-second session. All these stimulation sessions were always delivered at 100% of RMT for each participants.

We also recorded the post-stimulation clinical parameters of all participants on the Y-BOCS, HAM-A, HAM-D, and CGI scales at the end of 3 weeks of stimulation sessions and after 3 weeks of follow-up period (at 6 weeks from initial assessment). The rTMS side effects checklist was administered at the end of sessions for rating adverse effects. The data thus collected was tabulated and statistically evaluated using Statistical Package for the Social Sciences (SPSS) version 20. Chi-square test and Fischer’s exact test were used for analyzing categorical data, and t-test was used to analyze continuous data. A mixed-design ANOVA was used to compare Y-BOCS, HAM-D, and HAM-A scores between the groups.

## Ethical considerations

A written informed consent had been obtained for the study from all the subjects. The study started after getting ethical clearance from the Institutional Ethical Committee (**Approval number- 69/IHEC/2024)** and scientific clearance from the Institutional Scientific Committee.

## Results

**Figure 1.**
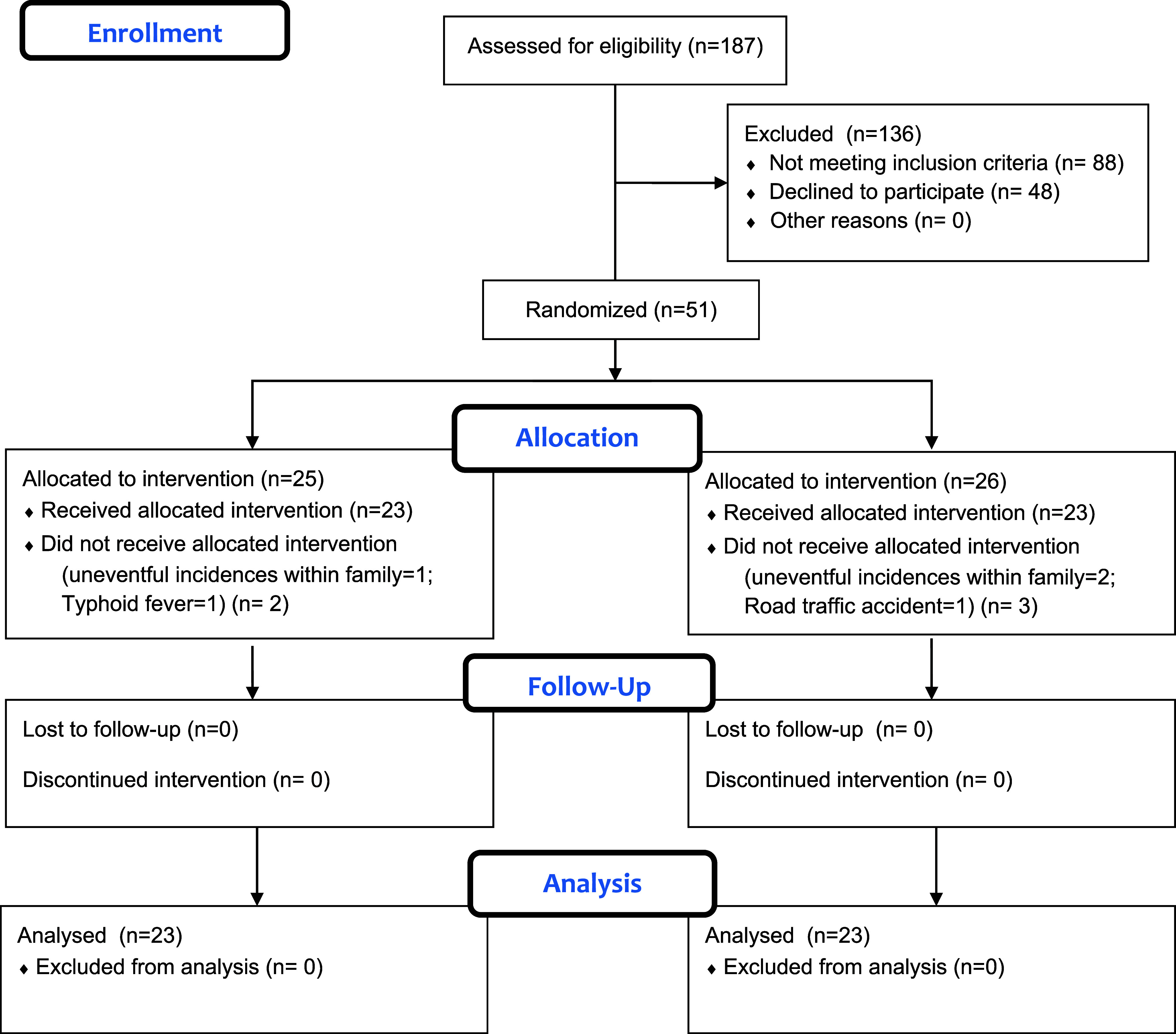
CONSORT 2010 flow diagram showing recruitment of patients.

A total of 187 patients with OCD were screened; amongst them, 51 patients fulfilling the inclusion and exclusion criteria were recruited for the study—25 patients in the low frequency rTMS group and 26 patients in the continuous theta burst stimulation (cTBS) group. Out of which 2 patients from the low-frequency rTMS group and 3 patients from the continuous theta burst stimulation group dropped out of the study before initiation of therapy. The reasons for dropping out of the study were inability to come due to uneventful incidences within the family (n = 3) and due to typhoid (n = 1) and a road traffic accident (n = 1) (Figure 1).

Twenty-five (54.3%) patients were female, belonged to age group of less than 30 years, were married and rural based and 36 patients (78.3%) belonged to middle class socio-economic background. Most of the patients had no past history (n = 39; 84.8%) or family history (n = 31; 67.4%) of psychiatric illness. Course of illness was continuous in 39 (84.8%) patients (Table 1).

**Table 1.** Socio-demographic and Clinical Variables of Participants (N = 46)

Variable	Mean (SD)	Frequency (%)
Age (in years)	28.8 (7.4)	
Marital status (married)		25 (54.3)
Age of onset (in years)	22.1 (6.3)	
Family history of psychiatric illness (no)		31 (67.4)
Course of illness (continuous)		39 (84.8)
Duration of illness (in years)	6.6 (5.3)	
Duration of prior drug treatment (in years)	3.7 (5.3)	
Past history of psychiatric illness (No)		39 (84.8)

We compared various socio-demographic and clinical profiles of our patients of low-frequency rTMS (LF rTMS) and continuous theta burst stimulation (cTBS) groups, enrolled in the study. There was no statistically significant difference found between the 2 groups on all the socio-demographic and clinical profiles compared (Table 2). Hence, both the groups were comparable at baseline on all socio-demographic and clinical parameters.

**Table 2.** Socio-demographic Variables Compared Between the 2 Groups

Variable	LF-RTMS n/(mean/SD)	cTBS n/(mean/SD)	X^2^/t	*P*-value
Age (in years)	30.0(7.1)	27.5(7.7)	t = 1.1	0.2
Sex				
Male	11	10	0.08	0.7
Female	12	13		
Marital status				
Married	13	12	0.8	0.7
Unmarried	10	11		
Occupation				
Employed	7	6	0.1	0.7
Unemployed	16	17		
Residence				
Rural	11	10	0.08	0.7
Urban	12	13		
Age of onset (in years)	20.4 (6.0)	21.9 (6.8)	t = 0.2	0.7
Duration of illness (in years)	7.6 (6.9)	5.6 (2.8)	t = 1.3	0.1
Duration of prior drug treatment (in years)	4.6 (7.1)	2.7 (2.2)	t = 1.2	0.2
Course of illness				
Continuous Episodic	21 2	18 5	Fisher’s exact test	0.4
Duration of illness				
≤ 5 years	9	11	0.3	0.5
>5 years	14	12		
Family history of psychiatric illness				
Yes	9	6	0.8	0.3
No	14	17		

There is no significant difference (p > 0.05) in Y-BOCS, HAM-A, and HAM-D scores between the 2 groups at baseline, the third week, and the sixth week of assessment (Table 3). There is no significant difference (p > 0.05) in YBOCS (obsession) score between the 2 groups at baseline. But at the end of the third week, we see a significant difference in the obsession scores between the LF-rTMS and cTBS groups. But, this difference disappeared at the end of the sixth week of assessment.

**Table 3.** Comparison of Rate of Improvement on Various Rating Scores Between the Groups from Baseline to Follow-up Periods (N = 46)

Variable	LF-RTMS group (n = 23)	CTBS group (n = 23)	F value	p-value
	Mean ± SD	Mean ± SD		
**YBOCS**				
YBOCS-Baseline	26.8 ± 5.4	27.1 ± 5.9	0.033	0.857
YBOCS- week 3	14.9 ± 5.7	18.1 ± 6.1	3.357	0.074
YBOCS- week 6	17.3 ± 5.2	19.5 ± 6.1	1.673	0.204
**YBOCS (obsession)**				
YBOCS (obsession)-baseline	14.1(2.6)	14.7(2.8)	0.550	0.462
YBOCS (obsession)-week 3	7.8(2.6)	9.8(2.9)	6.180	**0.017**
YBOCS (obsession)-week 6	9.3(2.6)	10.9(2.8)	3.580	0.065
**YBOCS (compulsion)**				
YBOCS (compulsion) -baseline	12.7(3.2)	12.4(4.0)	0.078	0.781
YBOCS (compulsion)-week 3	7.0(3.2)	8.2(3.6)	1.351	0.251
YBOCS (compulsion)-week 6	7.9(2.8)	8.6(3.7)	0.448	0.507
**HAM-A**				
HAM-A baseline	16.3(6.6)	16.9(6.8)	0.069	0.794
HAM-A week 3	8.7(4.1)	9.7(4.4)	0.621	0.435
HAM-A week 6	11.4(4.9)	12.5(5.5)	0.493	0.486
**HAM-D**				
HAM-D baseline	15.8(3.5)	14.7(4.0)	0.849	0.362
HAM-D week 3	8.3(4.1)	9.5(4.4)	0.914	0.344
HAM-D week 6	10.2(3.4)	10.7(4.1)	0.180	0.674

Assessment of side-effect profiles of our patients reveals that 4 patients (17.3%) in the LF-rTMS group and 9 patients (39%) in the cTBS group reported transient headache. Some minor side effects (eg, scalp discomfort, anxiety, and tingling) were reported in 2 patients of LF-rTMS group and 3 patients of cTBS group.

## Discussion

In this study, we had chosen low-frequency rTMS and cTBS for the comparison purpose because both have inhibitory effect on cerebral cortex. Based on neurophysiological data, it is possible that diminished cortico-subcortical inhibitory phenomena and higher-than-normal cortical excitability thresholds are the cause of motor “intrusive” and repeated habits in OCD patients.^28^ The rationale behind selecting low frequency (1 Hz) rTMS or cTBS for this investigation was that it could potentially act as a dampener for the motor cortex hyperexcitability that has been found in these illnesses. Recently, a shorter-term theta-burst stimulation (TBS) method with potential long-term benefits was introduced. With respect to rTMS, cTBS is more in line with the biological rhythm and has the ability to enhance synaptic long-term potentiation induction.^29^ Hence, both stimulation methods are worthwhile to be included in the study for comparison.

In our study, we targeted the right dorsolateral prefrontal cortex (DLPFC) for OCD treatment due to its relevance in the pathophysiology of CSTC networks and superficial location, making it easily accessible to magnetic stimulation. In comparison to this study, all previous cTBS studies in OCD had selected presupplementary motor area (pre-SMA), bilateral supplementary motor area (SMA), orbitofrontal cortex (OFC) as the therapeutic target.^9^ ^,^^12^ ^,^^19^ ^,^^21^ ^,^^22^ In our study, both groups received a total of 15 stimulation sessions at 100% of RMT for 3 weeks duration. The LF-rTMS group was delivered a total of 1600 pulses per session. It was in concordance with the previous studies, which have used 1500 pulses per session.^30^ ^,^^31^

In our study, we recruited patients who have been maintained on stable doses of medications for at least 2 weeks, and no further changes in medications were allowed during the study period. This was done to prevent spurious influence of change in medications on psychopathology. Previous studies have also included failure to respond to at least 2 SSRIs or a full CBT trial as an inclusion criterion in their studies.^32^ ^,^^33^Another unique feature of this study was the time point at which TMS intervention was introduced. In this study, the add-on of TMS was done early in the course of treatment. Early augmentation in OCD using neuromodulation like TMS and tDCS is a recently emerging concept.^11^ ^,^^28^ Early augmentation by use of neuromodulation has been seen to be reducing the severity of psychopathology quickly when used as an early augmentation strategy, which might be helpful for the patients in resuming the functioning early.^11^ ^,^^28^ ^–^^31^ More so, the present study compares 2 different treatment protocols (one with LF rTMS taking a longer duration and one with cTBS taking a short duration) targeting the right DLPFC (which is relatively less studied in comparison to the left DLPFC), which gives more uniqueness to this study.

The socio-demographic and clinical profiles of our patients of the LF rTMS and cTBS groups enrolled in the study are not significantly different. It gives a suitable platform for comparing the LF rTMS and cTBS. The right DLPFC is a relatively less explored target of TMS and TBS in comparison to other potential target areas (left DLPFC, SMA, pre-SMA, and OFC) in OCD. Emerging evidence suggests that there is reduced resting-state functional connectivity of the right DLPFC with the right middle temporal gyrus and right ventral anterior cingulate cortex.^34^ cTBS targeting the right DLPFC modulates the impulsivity level.^19^ These findings indicate the possible important implication of the right DLPFC in OCD.

There was an overall significant improvement in obsessive-compulsive symptoms in all our study population. When we tried to see the difference in the rate of improvement between the 2 groups, there was no statistically significant difference observed, except that at the end of the third week we saw a significant improvement in the obsession score in the LF-rTMS group (t = −2.4, p = 0.01) in comparison to the cTBS group. But this difference disappeared at the end of the sixth week of assessment. There is no study that head-to-head compared the efficacy of LF rTMS and cTBS targeting the right DLPFC in the management of OCD. A study by Khedr et al. (2022) found that LF rTMS targeting the right DLPFC and right OFC is superior to sham TMS in the treatment of OCD.^35^ A recent study using cTBS intervention over right OFC found that after 10 sessions of cTBS there is improvement in depressive symptoms; however, the improvement of OCD symptoms is not significant.^36^

There was overall significant improvement in anxiety and depressive symptoms in all our study population, when we tried to see the difference in the rate of improvement between the 2 groups, no statistically significant difference was observed.

In a study by Dutta et al. (2023), intensive cTBS over the orbitofrontal cortex (OFC) area had been shown to have clinically significant improvements in anxiety and depression symptoms and global severity in OCD patients.^32^ They concluded that improvement in anxiety symptoms could be due to modulations of state-dependent dysregulation in OCD. Our study findings are in line with the previous literature in this regard.

Many studies support the opinion that theta burst stimulation (TBS) over the left dorsolateral prefrontal cortex is an efficient treatment for major depressive disorder (MDD), which can significantly improve depression of MDD patients.^37^ ^,^^38^ Thus, the improvement in the depressive symptoms demonstrated in our study may be independent of the improvement in OC symptoms and may have different mechanisms altogether.

In contrast to our study, previous randomized placebo-controlled cTBS in OCD trials conducted by Guo et al. (2022)^39^ and Harika-Germaneau et al. (2019)^40^ reported that there was no statistically significant difference in treatment efficacy between cTBS and placebo treatment over the bilateral SMA and pre-SMA areas respectively. Our study used a higher intensity (100% RMT) and right DLPFC area hence, obtained positive results.

## Limitations

A sample size of 46 patients appears to be a small sample size. The representativeness of the sample was recruited from a single center in Eastern Uttar Pradesh and therefore limits the generalizability of the results of this study. A future study with a higher sample size from multiple centers will help us to generalize the findings of the present study. Similarly, the follow-up period was short. A long-term follow-up period will be able to give an idea about the sustainability of the TMS effect. The number of sessions delivered was only 15. More number of sessions is expected to give a different outcome. Another limitation is the absence of a neuronavigation technique to target the DLPFC. Moreover, the 2 procedures used in the study would not guarantee a perfect blinding, as a patient might be aware that LF-rTMS lasted for 28 minutes while cTBS only lasted for 40 seconds. Considering the limitations of the study, these findings need to be interpreted with caution with further research in a larger population, using additional imaging modalities, may further substantiate the findings.

## Conclusion

cTBS was found to be safe and had comparable short-term efficacy to traditional LF- rTMS for the treatment of OCD in routine clinical practice. The improvement in the obsessive-compulsive symptoms with cTBS and LF- rTMS over the right DLPFC is in line with available LF-rTMS and cTBS in OCD literature. The improvement in anxiety and depressive symptoms may add to the therapeutic advantage of adjunctive LF-rTMS and cTBS in OCD. The remission achieved by both treatment modalities in OCD patients was not sustained for a longer duration and appears to have faded with time.

## Data Availability

Data are not publicly available. Data will be made available on reasonable request from the corresponding author/ first author.
